# Electromechanical Properties of 3D-Printed Stretchable Carbon Fiber Composites

**DOI:** 10.3390/mi13101732

**Published:** 2022-10-13

**Authors:** Teemu Salo, Donato Di Vito, Aki Halme, Jukka Vanhala

**Affiliations:** Faculty of Information Technology and Communication Sciences, Tampere University, 33720 Tampere, Finland

**Keywords:** stretchable electronics, 3D printing, carbon fibers, electromechanical testing, strain sensor

## Abstract

The addition of fillers has been implemented in fused filament fabrication (FFF), and robust carbon fillers have been found to improve the mechanical, electrical, and thermal properties of 3D-printed matrices. However, in stretchable matrices, the use of fillers imposes significant challenges related to quality and durability. In this work, we show that long carbon staple fibers in the form of permeable carbon fiber cloth (CFC) can be placed into a stretchable thermoplastic polyurethane (TPU) matrix to improve the system. Four CFC sample series (nominally 53–159-µm-thick CFC layers) were prepared with a permeable and compliant thin CFC layer and a highly conductive and stiff thick CFC layer. The sample series was tested with single pull-up tests and cyclic tensile tests with 10,000 cycles and was further studied with digital image correlation (DIC) analyses. The results showed that embedded CFC layers in a TPU matrix can be used for stretchable 3D-printed electronics structures. Samples with a thin 53 µm CFC layer retained electrical properties at 50% cyclic tensile deformations, whereas the samples with a thick >150-µm CFC layer exhibited the lowest resistance (5 Ω/10 mm). Between those structures, the 106-µm-thick CFC layer exhibited balanced electromechanical properties, with resistance changes of 0.5% in the cyclic tests after the orientation of the samples. Furthermore, the suitability of the structure as a sensor was estimated.

## 1. Introduction

Additive manufacturing (AM) is widely used in several manufacturing sectors. Fused filament fabrication (FFF), especially, has advantages such as simplicity and cost-effectiveness [1]. For example, strain sensors [2], multiaxial force sensors [3], and batteries [4] have already been fabricated with this single-step FFF process. FFF can also be adopted in the textile field by printing plastics directly on textile substrates [5] or by printing the whole textile composition [6]. Moreover, it has been demonstrated that FFF with deformable plastics and substrates can be used in manufacturing stretchable electronics, which can be further laminated on textiles for wearable electronics [7].

Recently, 3D-printed stretchable and wearable electronics have gained more attention, and rigid [4] and stretchable wearables [8] have been successfully fabricated. Still, stretchable electronics that are practically integrable into clothing have not yet been manufactured via 3D printing. For integrable stretchable and wearable electronics, carbon-filled polymers are an especially promising alternative for the creation of mechanically complex and thermally and electrically conductive structures [2,9]. These polymers can be modified using carbon-based additives with different form factors and dimensions, such as carbon fibers [10], carbon nanotubes (CNTs) [2,3,9], carbon black [11], graphene [12], and others [13], with a wide variety of outcomes in terms of properties such as strength, thermal and electrical conductivity, piezoresistive behavior, and many others [14,15,16]. These modified materials enable the fabrication of sensors, wearables, and other end-products, e.g., by providing higher strength, fire retardancy, or electrical properties. However, FFF polymers with fillers generally require a high nozzle diameter to prevent clogging, decreasing the printing quality [2] and causing highly anisotropic printing results [17]. Fillers also rapidly increase the Young’s modulus of the polymers, leading to a trade-off between stiffness and conductivity [2].

In FFF polymers, the shape and size of carbon fillers influence the formation of the fillers’ conductive network, percolation threshold, and overall conductivity. For example, when the results from previous studies are converted into conductivity values, acrylonitrile butadiene styrene (ABS) filament consisting of 15 wt% nano-scale carbon black has 0.025 S/m conductivity [11], and 5.6 wt% graphene flakes with a lateral size of 3–5-µm [12] provide 0.001 S/m. CNTs were mixed into thermoplastic polyurethane (TPU) filaments (with a CNT content of 4 wt%) of in 9.5 nm diameter and 1.5 µm in length, providing 32 S/m conductivity after the 3D-printing process (with a 0.6 mm nozzle) [2]. Furthermore, Tzounis et al. blended TPU and CNTs (5 wt%, 9.5 nm diameter, 3.0 µm length), which resulted in higher conductivity, approximately 100 S/m, but this required a less accurate 0.8 mm nozzle [17]. Spoerk et al. 3D-printed polypropylene (PP) filaments filled with short carbon fibers (7 µm in diameter and 250 µm in length) at proportions of up to 10 wt% with a 0.6 mm nozzle [18] for thermally conductive structures. Even longer millimeter-scale fibers can be added during the filament manufacturing process, but these are chopped to the micrometer-scale during the process [10].

Furthermore, continuous carbon fiber filaments are used in FFF by feeding them into molten-state polymers during extrusion [19,20], increasing an object’s carbon fiber content. However, this process requires a larger nozzle [19,20]. Another alternative is to impregnate carbon fiber filaments before printing, enabling more complex [21] and precise [22] printing with filaments.

Feeding carbon fillers and carbon filaments through a 3D printer’s nozzle makes them compatible with readily available FFF printers. Furthermore, carbon semi-products, such as laminates and inks, can be integrated into the 3D printing process semi-automatically or automatically by pausing the process. Carbon fiber sheets can be laminated on top of objects to form durable and lightweight composite structures [23] or inside them to address porosity and layer adhesion issues [13]. Moreover, integrated carbon fiber tows can be used to monitor a matrix’s structural health via the tows’ resistance changes [24]. Other electrical and thermal properties can be created by spray-depositing the 3D-printed surface with CNTs, and a 19-nm layer thickness on the smoothened surface is possible [25,26]. Furthermore, the direct ink writing (DIW) method can be combined with FFF to print carbon black ink electrodes for 3D printed supercapacitors [27]. The FFF process even allows the manual or automatic integration of printed circuit board (PCB) components inside an object [8], which, along with other placement methods, enables versatile 3D-printed electronics.

In this study, stretchable and wearable 3D-printed electronics components were made by adding sparse carbon fiber cloths (CFCs) inside a TPU matrix made with FFF. The advantages of this process are that CFCs with mechanical and electrical properties were (1) integrated inside the matrix without cavities or other 3D printing design modifications, and (2) adhesives were not required for their placement—it was sufficient to change the general 3D printing settings. Furthermore, CFCs embedded in this way inside the structure (3) do not decrease the adhesion between the TPU layers, and (4) they can be cut into different shapes, which can be used as functional elements in fabricating stretchable and wearable electronics. Furthermore, integrated CFCs can create (5) more isotropic and detailed objects than those created through FFF with carbon fiber filaments.

To the authors’ knowledge, permeable CFC has never been used to improve the electrical and mechanical properties of FFF objects. The measured properties of CFC compare favorably to those of carbon-filler filaments and can be used to provide stretchable and conductive composite matrices. The obtained results prove that matrices with CFC can sustain large numbers of deformation cycles with minimal changes in their resistance behavior, thanks to the combined mechanical and adhesion properties of the materials involved. We studied the characteristics and advantages of this new method by conducting quasi-static and cyclic electromechanical tests, which we further analyzed using digital image correlation (DIC) techniques to attain information about the local deformation field in the samples. Finally, the properties of the structure for sensor applications were estimated.

## 2. Materials and Methods

CFCs with centimeter-scale fibers are traditionally used in composite manufacturing to increase the Young’s modulus of materials with a minimal increase in density, i.e., to improve the specific modulus of materials. As well as mechanical features, CFCs are thermally and electrically conductive, thus having the potential to be used in wearable electronics. The stretchability that wearable electronics require was achieved by combining sparse CFCs and a highly stretchable TPU matrix. Single pull-up tests were first used to study the mechanical properties of the CFC matrices. Then cyclic tensile tests were conducted to measure the electromechanical features. Finally, the samples’ behavior was further analyzed with DIC.

### 2.1. Composition and Preparation of Samples

The CFCs used in this work were provided by ACP Composites [28], and the reported average single carbon fiber length was 25.4 mm. The carbon fibers were processed from polyacrylonitrile (PAN) to promote their electrochemical properties [29]. CFCs with two grades of nominal thicknesses, 0.0021” (53 µm) and 0.006” (153 µm), were tested. Thin CFCs were also tested as two- and three-layered plies to further improve their electrical conductivity. The single-layer CFCs and the two- and three-layered plies were laminated to make them flat and fixed together for the 3D printing process, which can decrease the nominal thicknesses of CFCs. Furthermore, plain zero samples without CFCs were tested for comparison. The composition of the carbon fiber and its nominal thickness in the electromechanical samples are presented in Table 1.

A TPU filament was used as a 3D-printed backbone for the fabricated structure. TPU is a widely used material in 3D printing and stretchable electronics because of its high deformability and stability [2,3,7]. For example, TPU-based stretchable films have also been used in wearable and printed electronics in combination with screen-printable conductive silver inks. Using TPU filaments is a convenient choice for developing 3D-printed stretchable and wearable electronics because of the ease of integration in these types of systems. Blue Ultimaker TPU 95A filament (Ultimaker B.V., Utrecht, The Netherlands) by Ultimaker B.V. (nominal diameter: 2.85 mm) was used in the tests, as it is more reliable to 3D print compared to the thinner 1.75 mm diameter FFF filaments, which are prone to bend and jam during 3D printing.

A commercial Ultimaker S5 FFF printer (Ultimaker B.V., Utrecht, The Netherlands) with an official air management unit accessory, with the Cura slicer program (version 4.4.0, Ultimaker B.V., Utrecht, The Netherlands) from Ultimaker B.V., was used in the 3D printer setup. The nozzle diameter was 0.4 mm, and the layer thickness was 0.15 mm. The nozzle temperature was 240 °C and the bed temperature was 60 °C, enabling good adhesion on a clean glass building plate. The printing speed was 25 mm/s, and the cooling fan was off. The number of walls was two, the infill ratio was 100%, and the infill shape was 45° lines. Furthermore, the infill was printed before the walls so that the CFC piece was smoothly fixed on the printed surface. As well as the typical printing settings, a script was added in the middle of the printing program to pause the printing automatically to manually insert the CFC piece. Furthermore, a prime tower feature was used before printing on top of the applied CFC piece to avoid uncontrolled leaking of the molten TPU from the nozzle onto the sample.

**Figure 1:**
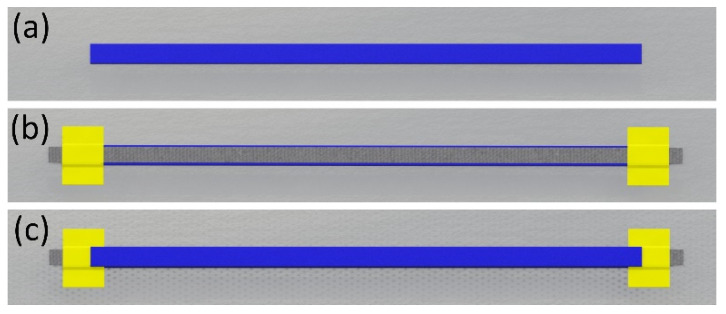
Preparation steps of the samples as modeled in Solidworks: (**a**) 3D printing of the sample’s bottom half-layer, (**b**) placement of the CFC piece with the use of Kapton tape, and (**c**) continuing the 3D printing until the sample was ready.

FFF was used to fabricate samples, of which the target dimensions were 10 mm wide, 200 mm long, and 1 mm thick. In the middle of the samples, an 8-mm-wide and 230-mm-long piece of CFC was placed longitudinally. CFCs were cut in the machine direction orientation (MDO), their loose ends serving as electrical contacts. Then, the CFC was fixed with two strips of Kapton tape to avoid using an adhesive and to reinforce the electrical contacts. The placed CFCs affected the samples’ dimensions, measured with a digital Vernier caliper with ± 0.01 mm measurement resolution. The sample preparation steps, drawn in Solidworks 3D design software (version 2021, Dassault Systèmes SolidWorks Corporation, Waltham, MA, USA), are presented in Figure 1.

### 2.2. Electrical Measurement of the Samples

The CFC pieces were electrically conductive. Based on the amount of carbon fibers they contained, they provided different levels of electrical conductivity in the 3D-printed matrix. In the sample preparation, the resistance of the CFC pieces was measured twice: before their placement inside the sample and after the 3D printing process. The resistance was measured using a Fluke 183 multimeter (Elfa Distrelec Oy, Helsinki, Finland) by firmly pressing the multimeter probes on the CFC pieces to achieve a stable reading (<5 Ω variation). The distance between the probes was 220 mm. The comparability of the results to those of previous studies was enabled by converting resistance to conductivity. The conductivity was calculated with the equation

*σ* = 1/((*RA*)/*L*), (1)

where *σ* is the conductivity in S/m, *R* is the resistance in Ω, *A* is the cross-sectional area of CFC in m^2^, and *L* is the distance between probes in m. The cross-sectional area was calculated based on the nominal width and thickness of CFCs, as reported in Table 1. Note that the conductivity here does not refer to the carbon fiber’s conductivity but to the average conductivity of the macroscopic sample’s CFC material.

### 2.3. Mechanical Tests

The mechanical behavior of the 3D-printed samples and the effect of the quantity of the integrated CFCs on the failure behavior was evaluated with an ESM303 tensile tester (Mark-10 Corporation, Copiague, NY, USA), equipped with a 500 N load cell. The distance of the clamps was 50 mm; the movement speed of the upper clamp was 25 mm/min. From each sample series, two samples were elongated 50% (25 mm) with a single pull-up test.

Because clothing-integrated stretchable and wearable components endure thousands of stretching cycles during their lifetime [30], cyclic tensile loading was chosen as a more realistic testing method. The cyclic electromechanical behavior of the samples was tested with the simultaneous use of the tensile tester and a custom-built resistance measurement system (Tampere University, Tampere, Finland). In the cyclic electromechanical tests, the movement speed of the upper clamp was 240 mm/min.

A custom-built resistance measurement system was constructed using an Arduino Uno board. The system used Arduino Uno’s 10-bit AD converter for two-wire measurements to calculate real-time voltage values over the samples, which were converted to resistance values and recorded. The system had three measurement channels that used 3470 Ω resistors as a reference. Based on the reference resistor values, the system’s accuracy was ±3 Ω. A threshold of 3000 Ω was used to indicate the total sample failure. The probes of the resistance measurement system were fixed to the samples’ contacts with anisotropic conductive adhesive film (ACF), and were further clamped to ensure stable electrical connections.

Since the conducting material’s structure is not homogeneous but is rather an interconnected network of fibers with small contact points between the fibers, the current density may influence the sample’s resistance. Nevertheless, no such effect was observed with the low (less than 100 mA) measured DC currents. With higher frequencies (in MHz range) or high currents (several A), the influence of current density on the resistance would probably be observed, in alignment with previously reported results [31].

**Figure 2:**
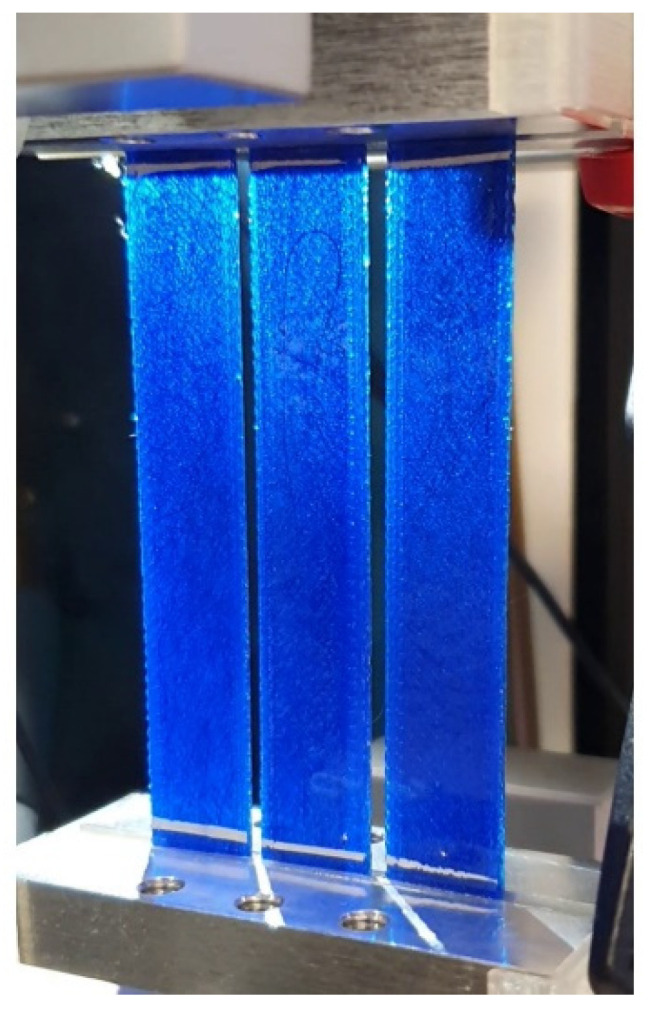
Clamped cyclic test samples in the ESM303 tensile tester. The samples were inspected with a background light, which revealed the integrated CFCs inside the 3D-printed TPU matrix.

In the cyclic tests, three samples from the same category were fixed together in the 50-mm-wide clamps and simultaneously stretched 10,000 times. During testing, the tensile tester measured the average force and displacement of three samples, and the resistance measurement system measured each sample’s resistance. For every sample series in Table 1, five degrees of tensile deformations were tested (10%, 20%, 30%, 40%, and 50%), and 25 cyclic tests were conducted. Figure 2 presents the clamped cyclic test samples with background light. After the cyclic tests, a strain sensor test was carried out with the cyclic test setup and a previously tested (50% elongated) cyclic test sample with a 153 µm nominally thick carbon fiber layer. The sample was elongated repeatedly up to 10% with a speed of 0.5 mm/min.

The cyclic test analysis was performed using Matlab (version R2021b, MathWorks, Inc., Natick, MA, USA). Maximum and minimum resistance values during cyclic loading were extracted from the test data by averaging the steady-state results obtained after the first thousand cycles, and a high variation in the sample resistance was observed, presumably due to fiber reorientation in the CFC layer. The change in the samples’ resistance was then calculated based on the two parameters extracted above. Furthermore, the average change in the resistance of the cyclically loaded samples was calculated by dividing the testing time into ten cycle periods and calculating the average resistances of each period. After that, the average resistance data from the last 5000 cycles, with which the increase in resistance versus the cycle could be linearized, were fitted using a linear relationship through the polyfit Matlab function (*n* = 1) to obtain the average resistance change per cycle.

### 2.4. DIC Analyzes

To further analyze the electromechanical properties of the samples, DIC was used for separate cyclic tests to inspect surface deformations of the samples in 100 early cycles, in which the largest resistance changes typically occur. The surfaces of the samples were studied and compared in the first, 50th, and 100th cycles. For this purpose, the deformation of the samples during loading was recorded using the stereo 3D DIC imaging system 3D StrainMaster Compact 5M (LaVision GmbH, Göttingen, Germany) and analyzed using DaVis software (version 10.2.1, LaVision GmbH, Göttingen, Germany). The strain measure used through the DIC analyses—and thus the one shown in the figures in this work—was the logarithmic (Hencky) strain. For DIC, the speed of the upper clamp was 50 mm/min, and the maximum elongation of the samples was 50% (25 mm).

The CFC pieces were studied with DIC without the 3D-printed TPU matrix to observe the mechanical limits of the fabricated CFC pieces more closely. In these samples, each CFC piece was laminated between two clear TPU films (Platilon U 4201 AU by Covestro). The thickness of the films was 100 μm, width 10 mm, and length 200 mm. The film was transparent and notably more deformable under the same load levels compared to the 1-mm-thick 3D-printed TPU matrix, enabling the evaluation of the deformation of the CFC pieces.

## 3. Results

### 3.1. Preparation of the Samples

The dimensions of the designed samples were 10 mm in width, 1 mm in thickness, and with a cross-sectional area of 10 mm^2^. Table 2 shows the measured average dimensions of the sample series that underwent cyclic electromechanical testing, classified by amount and type of reinforcement. The sample series contained 15 parallel samples.

The area of the unreinforced sample series was 1.5% larger than the target dimensions. With one layer of thick CFC, the average area increased by 4.9%. With 1–3 layers of thin CFC, the average area increased by 4.1%, 5.6%, and 8.3%, correspondingly.

**Figure 3:**
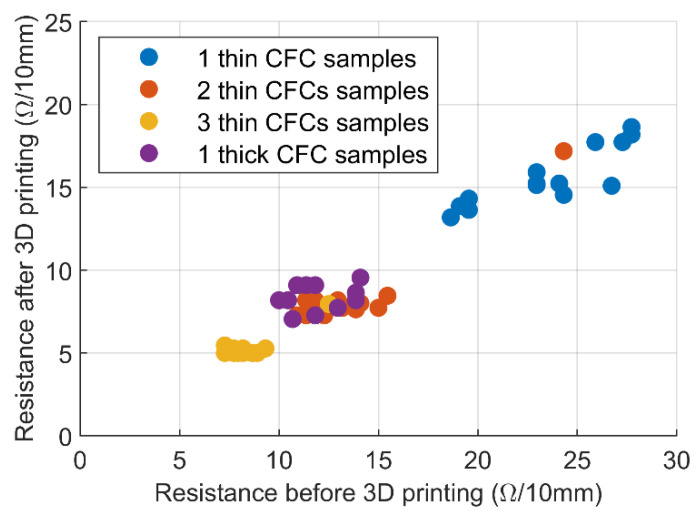
CFC layers’ resistance per 10 mm before and after the 3D printing process. The sample series contained 15 parallel samples.

Figure 3 shows the resistance measurements of the CFC pieces of the samples before 3D printing, compared with the same measurements after the process. From these data, it is evident that CFCs’ electrical performance improved after their integration into the 3D-printed matrix, since their resistance decreased. This differs, for example, from the currently used carbon filler filaments, which have better electrical properties before 3D printing. The resistance of the samples with thick CFC decreased by 27% after 3D printing, making the average conductivity of the sample series approximately 1000 S/m. In the sample series with one thin CFC, the resistance decreased by 33%, whereas the sample series with two thin CFCs and three thin CFCs exhibited a 38% decrease in resistance. The calculated conductivity of all thin CFC sample series was at the level of 1500 S/m, which is not a drastic variation, despite their differing thicknesses and several interfaces.

In addition to the decrease in the resistance, Figure 3 shows the scattering of the resistance values in the CFC pieces. The series with one thin CFC showed the highest dispersion of results, which decreased when more CFC plies were laminated on each other. Furthermore, although the thickness of the sample with one thick CFC and that of the sample with three thin CFCs samples were nominally similar, the resistance values and scattered results of the series with one thick CFC were at the level of the series with two thin CFCs.

### 3.2. Mechanical Tests

**Figure 4:**
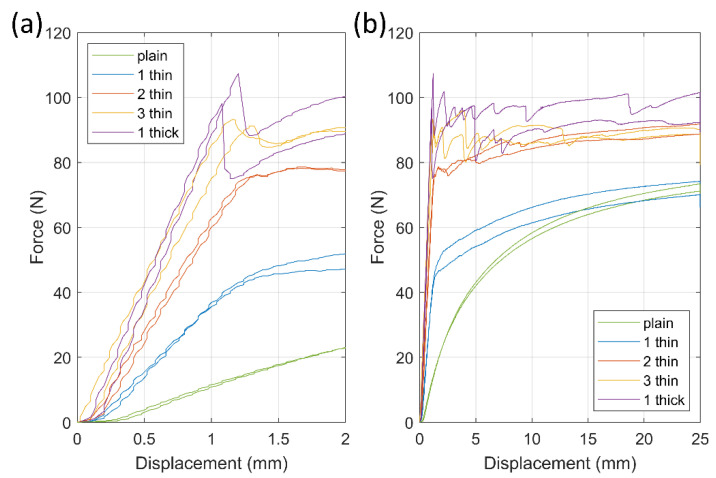
Force–displacement curves of the 3D-printed samples. (**a**) Results focusing on the initial 2 mm displacement (4% elongation), presenting the increase in stiffness caused by CFCs, (**b**) the samples’ total displacement was 25 mm (50% elongation).

First, the samples were elongated by 50% in the single pull-up tests; the results are shown in Figure 4. In Figure 4a, it is possible to note that in the initial elastic phase, before any crack formation in the samples, the increased amounts of CFCs added to the samples resulted in larger slopes in the elastic region of the force-displacement curve. The high amount of CFCs affected the elastic phase end, with the deformation developing into a permanent plastic phase, the transition area becoming irregular, and random force drops appearing. After the elastic phase, the decline in the force of the samples with one thick CFC was about 20 N (20%) and it was approximately 5 N (6%) in the samples with three thin CFCs. Furthermore, Figure 4b shows that the force results of the samples approached certain levels, with the force of the plain samples and the samples with one thin CFC approaching 70 N. In the samples with two thin CFCs and with three thin CFCs, the force levels were around 90 N, whereas the force in the samples with one thick CFC varied between 90 and 100 N.

**Figure 5:**
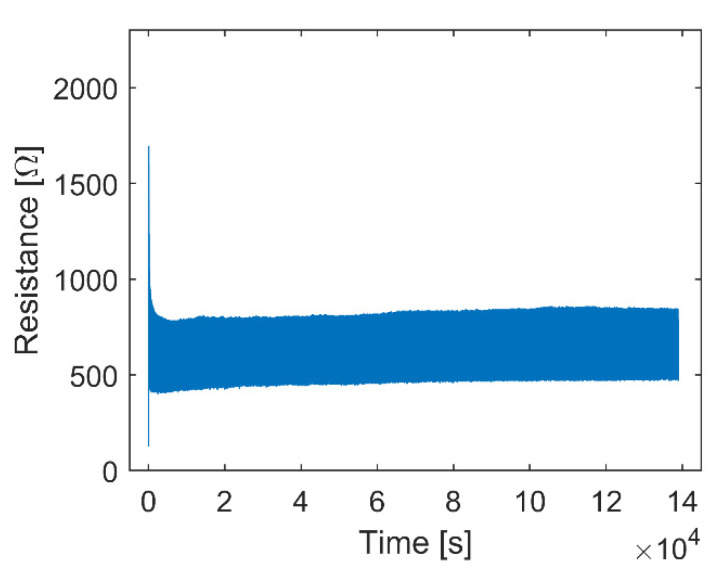
Raw data of a sample from the sample series with two thin CFCs, elongated 50% 10,000 times. The data show the high increase in resistance in the first cycle and the decrease in resistance and stabilization that occurred within a few cycles.

**Figure 6:**
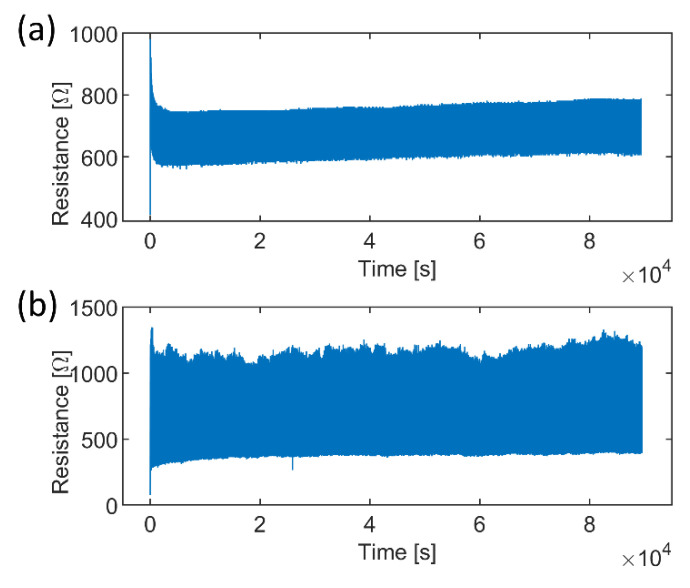
Cyclic behavior comparison of the samples elongated 30%. (**a**) The sample with 1 thin CFC exhibited repetitive electrical behavior in the 600 Ω–800 Ω range, whereas (**b**) the sample with 3 thin CFCs exhibited varying properties in the 350 Ω–1250 Ω range.

After the single pull-up tests, the samples’ long-term durability was examined through cyclic tensile tests. The raw data concerning resistance over time showed that three phases could be identified for each cyclic test. Data from one sample from the sample series with two thin CFCs (50% elongation) are shown in Figure 5. Initially, there was (1) a cycle in which a high increase in the sample’s resistance was observed, followed by (2) a few more cycles with decreasing resistance values, which finally (3) stabilized to a specific level. In phase 3, the resistance behavior over the cycle was predictable and stable, or it varied unpredictably and accumulated damage, as shown in Figure 6.

The samples’ resistance varied differently depending on the CFC layer’s thickness, structure, and tensile deformation level. The samples with a thin CFC layer and a low degree of elongation were more likely to have a more stable resistance variation in phase 3 (Figure 6a) than the samples with a thick layer of CFCs and high elongation (Figure 6b). In some cases, the resistance values in phase 3 exceeded the measurement limits of the test setup (3000 Ω), indicating the electrical failure of the sample.

In addition to the resistance behavior, the samples with different CFC layers exhibited different elongation and failure behavior (Figure 4). The samples with 1–2 layers of thin CFCs elongated uniformly, but those with a stiffer CFC layer elongated more locally. Thinner CFC layers tended to show delocalized damage and deformation along the whole sample through the TPU matrix; in the thicker samples, the deformation was localized where the CFC layers started forming cracks, leading to high local strains in the matrix, greatly affecting the samples’ electromechanical properties.

**Figure 7:**
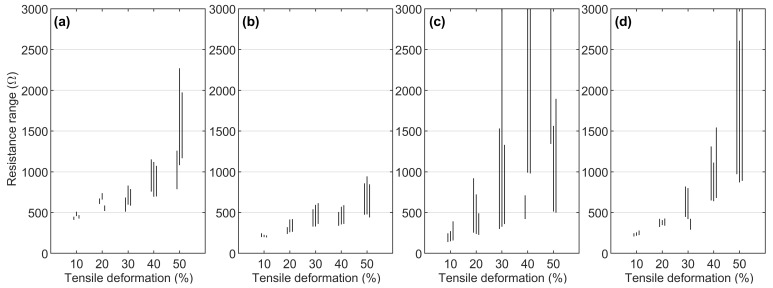
Resistance range lines of cyclic sample series, where three parallel samples are grouped together. The bottom and top endpoints of the lines show the minimum and maximum resistance values of the samples: (**a**) series with 1 thin CFC, (**b**) series with 2 thin CFCs, (**c**) series with 3 thin CFCs, and (**d**) series with 1 thick CFC.

Figure 7 shows the resistance ranges of the cyclic sample series, with the electromechanical behavior of each measured sample regarding its resistance properties is displayed as a vertical line. The results of the three parallel samples are grouped, and the endpoints of the lines show the maximum and minimum resistances, whereas the line’s length displays the resistance change during stable cyclic loading. As depicted in Figure 7a, the sample series with one thin CFC showed a moderate, gradual increase in its resistance range before the final 50% deformation level, which increased the samples’ resistance range and variation. Figure 7b shows the modest resistance behavior of the series with two thin CFCs, with similar resistance values at the 30% and 40% deformation levels; at the 50% deformation level, the resistance range was less than 500 Ω.

As illustrated in Figure 7c, the stiff series with three thin CFCs showed more scattered results regarding the maximum resistance and resistance range values, whereas the minimum resistance level still exhibited a recognizable gradual increase. Finally, as shown in Figure 7d, the series with one thick CFC behaved similarly to the series with one thin CFC. However, the resistance was lower at the 10–20% deformation levels because of the greater thickness of the CFC layer. Conversely, this sample series showed higher resistance changes at the deformation levels of 40% and 50% because of its stiffness.

**Figure 8:**
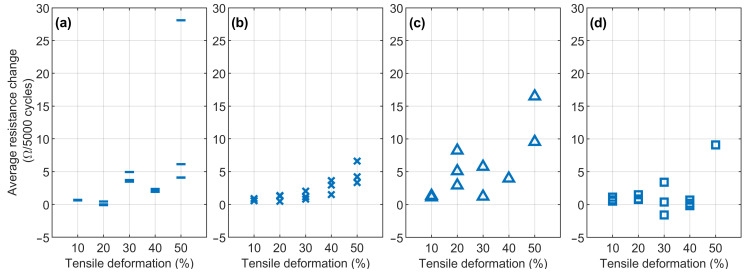
Average resistance changes between cycles in the last 5000 test cycles: (**a**) series with 1 thin CFC, (**b**) series with 2 thin CFCs, (**c**) series with 3 thin CFCs, and (**d**) series with 1 thick CFC.

Like Figure 7, Figure 8 shows the changes in the average resistance in the samples per 5000 cycles, which indicates how much the resistance changed in the stable cyclic deformation phase of the cyclic samples (previously defined as phase 3). The failed samples that reached the 3000 Ω failure limit in Figure 7 are absent from Figure 8.

In Figure 8a, the series with 1 thin CFC exhibited a change of approximately 5 Ω (1%) or smaller throughout 5000 cycles, except for a 50% tensile deformation level. The more random 50% tensile deformation level indicates the possible electrical limits of the sample series. In Figure 8b, the change in resistance increased as the tensile deformation increased; in the 10–30% tensile deformations, the average resistance change was always lower than 3 Ω (0.5%). At the 40–50% level, the resistance change was still small, but the low resistance range in Figure 7b increased the percentual resistance change and the results were approximately 5 Ω (lower than 1%).

In Figure 8c, the scattered resistance values show that the samples with three thin CFCs were not deformed evenly. Moreover, several samples (one sample from the 30% deformation level, two from the 40%, and one from the 50% level) were removed as failures. Despite this, the samples’ percentual resistance change was lower than 1%; even with two unstable samples at 20% and 50% deformation levels, the change was approximately 2% and 1.5%, correspondingly.

Figure 8d depicts the results of the series with 1 thick CFC, which differed from the rest of the sample series in Figure 8 and which exhibited small negative resistance changes (lower than −0.3%) at the 30% and 40% tensile deformation levels. In the samples, the resistance decreased throughout the cycles, which can be explained, e.g., by the alignment of the carbon fibers. Furthermore, the average resistance changes were small at the 10–40% levels and were always less than 4 Ω (1%) due to the high thickness of the CFC layer. This, however, changed at the 50% deformation level, where the CFC layer’s stiffness was high enough (compared to one of the TPU matrices) that the sample continued accumulating damage, resulting in only one intact sample at the end of the cyclic test that showed an average resistance change equal to 10 Ω per 1000 cycles.

### 3.3. A Strain Sensor Demonstration

**Figure 9:**
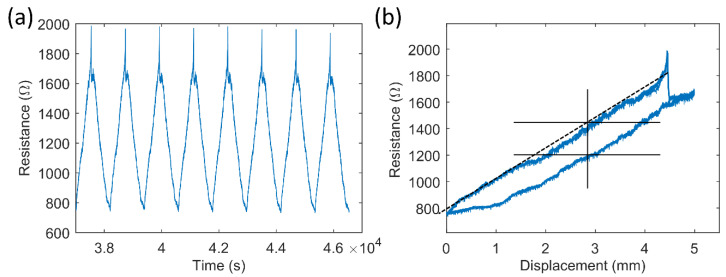
Sensor properties of tested cyclic test samples (153-µm-thick CFC, 10,000 cycles, 50% elongation, 240 mm/min speed). (**a**) Raw data with 10% elongation and 0.5 mm/min speed, and (**b**) a resistance-displacement curve of the first 4 cycles from (**a**).

Finally, the usage of 3D-printed samples with an integrated carbon fiber layer as a strain sensor was tested, with results shown in Figure 9. As an interconnection, the samples lasted well up to 50% elongation, but for sensor applications, a smaller 10% dynamic range was used after the initial 10,000 cycles, with a maximum elongation of 50%.

In the tests, rapid changes caused a dynamic error, and the response to a step change stabilized in about 200 s. The test in Figure 9a was carried out with a stretching speed of 0.5 mm/min and a maximum elongation of 10%. Figure 9a shows a linear increase in resistance, which is optimal for sensing applications. However, there was some drift in the output signal, similarly to what can be seen in the cyclic test in Figure 5. The drift stabilized after about 100 cycles.

As shown in Figure 9b, the practical dynamic range of the sensor was 750 Ω–1800 Ω. The system shown in Figure 9b was not strictly linear. The nonlinearity error and noise were <5% of the full scale. The sensor’s output after the structure stabilized was repeatable, and the nonlinearity could be compensated for using software.

Figure 9b also shows hysteresis and sensitivity. The maximum difference during the hysteresis cycle was 220 Ω, which corresponds to ~10% of the full scale. The sensitivity of the sensor structure was ~200 Ω/mm.

### 3.4. DIC Analyses

**Figure 10:**
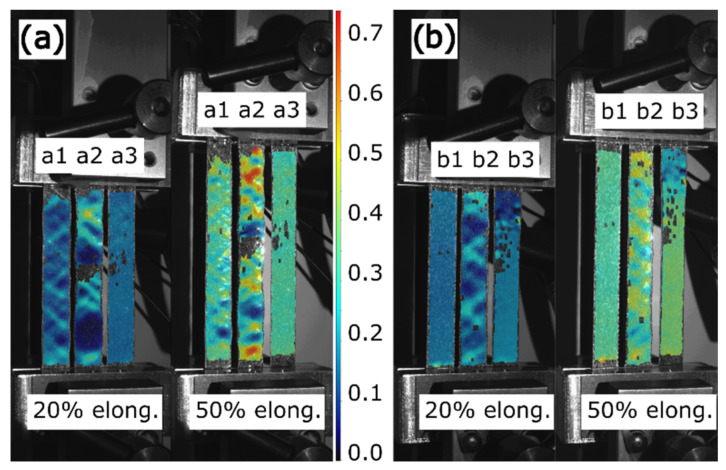
DIC *y*-axial Hencky strain results of 3D-printed samples in 20% and 50% elongations: (**a**) the a1 sample with 2 thin CFCs, the a2 sample with 3 thin CFCs, and the a3 sample with 1 thin CFC; (**b**) the b1 sample without CFCs, the b2 sample with 1 thick CFC, and the b3 sample with 1 thin CFC. The scale bar shows the level of local deformations compared to the initial undeformed phase of the samples.

The 3D-printed samples are also studied with DIC to identify local deformations to supplement the mechanical test results. The results of DIC analyses for the different sample series are presented in Figure 10.

Figure 10 shows that samples with different embedded CFC layers also exhibited different levels of local deformations. The non-reinforced plain sample (b1) and those with one thin CFC (a3 and b3) elongated uniformly without visual signs of deformation in the vertical direction. The stiffening effect of one thin CFC did not affect the overall surface deformation of the TPU matrix, nor did it create stress concentration areas. Instead, the sample with two thin CFCs (a1) showed multiple oblique failure bands that followed the shape of a 45° infill pattern over the entire length of the sample. Moreover, the high strain peaks observed here differed from the bulk level of deformations by only 20%.

The stiffest samples (a2 with three thin CFCs and b2 with one thick CFC) showed prominent failure bands, where failure was concentrated on the CFC layer. At 50% elongation, it is possible to note that some areas in the sample with three thin CFCs were almost undeformed (5%), whereas other areas exhibited axial deformation reaching over 70% due to the complete local failure of the CFC layer. The sample with one thick CFC also displayed a visible failure with a 40% deformation difference between the undeformed and failure areas.

**Figure 11:**
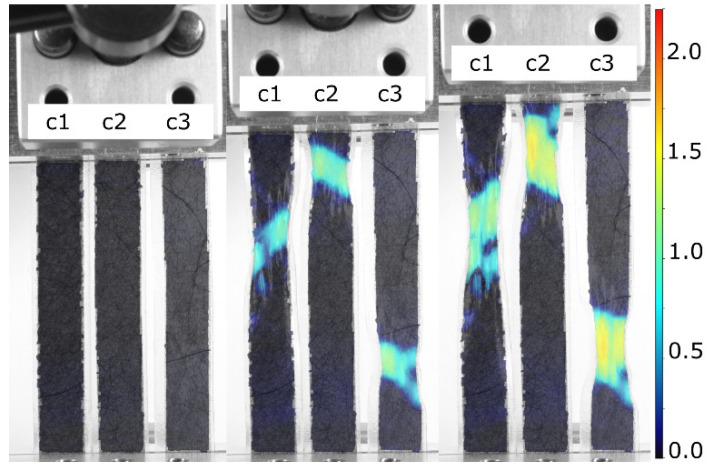
DIC *y*-axial Hencky strain analyses of the elongation of laminated CFC samples, where the c1 sample had 1 thin CFC, the c2 sample had 2 thin CFCs, and the c3 sample had 3 thin CFCs between the transparent TPU films. The deformation was mostly localized in the failure locations, whereas the rest of the sample exhibited almost no deformation under the load. The scale bar shows the level of local deformations compared to the initial undeformed phase of the samples.

As well as the 3D-printed samples, the deformation of CFC pieces embedded between thin and highly elastic TPU films is presented in Figure 11. In these cases, the stiffness of the CFCs and the TPU films differed considerably, making the CFC piece the load-carrying component and the TPU films only the binders of the CFC piece.

The DIC examination, shown in Figure 11, indicated that the failure of the CFC piece occurred at the same deformation level for samples with different thicknesses and structures. Generally, when a failure was localized at one point on the CFC piece, the elongation was focused on that area. For instance, in the highly elongated samples shown in Figure 11, the dark-colored CFC areas were elongated by less than 10%, whereas the elongation in the cracked areas could reach over 150%. The results showed that the highly deformable TPU films around the CFC piece did not distribute loads across the CFC piece and required a stiffer TPU matrix, which can be made through FFF.

## 4. Discussion

### 4.1. Effect of CFCs on the 3D Printing Process

Adding CFCs into FFF had both negative and positive effects on the process and the prepared object. The object’s dimensions changed when CFCs were added inside an object without a cavity (Table 2). The samples’ dimensions increased when the amount of CFCs increased since the carbon fibers replaced molten polymer on the layer, forcing it to flow elsewhere. However, when considering FFF’s general accuracy, the calculated volume increases were small, and these changes can be considered in the design phase.

In the preparation of the samples, integrating CFC pieces into the TPU matrix changed the CFC layer’s electrical properties. The 3D-printed molten plastic on the CFC piece compressed the fibers on the previously printed layer in the *z*-direction, improving the CFCs’ electrical properties by decreasing the resistance by about 30%. Furthermore, the shrinkage of the molten plastic during cooling may have compressed the CFC layers even more in the *x*- and *y*-directions, improving their electrical properties more. The measured sample series had different amounts of integrated CFC layers, and the series with one thin CFC (a nominally 53-μm-thick CFC layer) showed scattered resistance results, as shown in Figure 3. When the CFC layer’s nominal thickness was at least 100 μm, the sample’s final resistance value was more consistent and predictable. Moreover, the series with two thin CFCs series and that with one thick CFC had approximately the same measured electrical behavior despite their 50 µm difference in thickness, indicating that lamination and the inclusion of an interface between the two CFC plies even out the electrical properties of randomly oriented CFCs efficiently.

The conductivities of the thin and thick CFC pieces were about 1500 S/m and 1000 S/m, correspondingly. The lamination of two or three thin CFCs plies together did not change the conductivity despite its effect on the resistance values. Notably, the nominal thicknesses of the CFC plies were used in the conductivity calculations. In case of the possible compression of the plies, a lower thickness would mean even higher conductivity. Furthermore, in measuring the initial resistances (used for the conductivity calculations), the accuracy of the resistance measurements was affected by the contact resistance and surface topography of the permeable fiber matrices. The inaccuracy was the same in the case of the CFC pieces (<5 Ω). Nevertheless, sample conductivity obtained using this method was two orders of magnitude higher than that of the methods currently used to incorporate carbon particles into FFF-generated objects, which justifies the use of CFCs in FFF matrices.

Also notable is that adhesion of the TPU matrix and the CFC piece was solid, showing no traces of delamination. The CFCs were placed on the additive manufactured surface without adhesives, and the flowing molten TPU fixed the CFC pieces inside the matrix’s TPU infill structure. It is also likely that the molten TPU did not fully penetrate the thick CFC piece but still encapsulated the CFC layer inside the sample.

### 4.2. Electromechanical Behavior of the Samples

All the integrated CFC sample series were produced using the same 3D printing process, but their electromechanical properties varied. Independently, the CFC plies had different electrical properties because of their thicknesses and fabrication procedures, which, however, did not affect properties such as their elongation at break (Figure 11). Furthermore, considering the results of the plain TPU samples, one can claim that the samples’ electromechanical properties came from the unique combination of the 3D printed TPU and CFCs.

The cyclic tensile test samples exhibited three phases in their tensile deformations (Figure 4), in which the first cycles might be explained through the rearrangement of the carbon fibers in the material. During the first loading cycles, carbon fibers embedded in the 3D-printed material may experience high enough local deformation, which may break part of the conduction network; as this induces an increase in resistance, some of the fibers that are now free to move to rearrange themselves due to the highly directional deformation applied, thus creating new (and more stable) paths that lead to a substantial decrease in the overall resistance. However, this phenomenon is not fully explained and requires more analysis.

The third phase is related to the electromechanically stabilized area that is observed after the orientation of the fibers. The regularity of phase 3 is affected by the amount of tensile deformation, thickness, and the number of interfaces in the CFC piece. Moreover, phase 3′s erratic behavior commonly affects only the maximum resistance values per cycle, whereas the minimum value stays at the same level. In the series with one thin CFC, phase 3 was even, but the resistance range in the cycles was extensive. In the series with three thin CFCs series and that with one thick CFC, phase 3 was unstable because of increased damage accumulation in the CFC layer. The most promising was the series two thin CFCs, where phase 3 was stable, and the resistance varied only 500 Ω between the maximum and the minimum during cycling at 50% tensile deformation.

#### 4.2.1. Plain Sample Series and Series with One Thin CFC

In the plain sample series and that with one thin CFC, the deformations of the samples were determined by the properties of the TPU matrix. The plain sample series exhibited smooth force-displacement curves, as shown in Figure 4, which are typical for rubber-like materials. Adding one thin CFC into the matrix changed the curves, so that there were (1) linear elastic and (2) settling plastic deformation areas. The samples with one thin CFC elongated evenly, and their force approached the same level as that of the plain samples, meaning that the thin CFC influenced the samples’ initial stiffness and the TPU matrix carried a load further at higher elongations. The 53-μm-thin CFC’s minimal effect on the long-term deformation of the TPU matrix can also be seen in the DIC results in Figure 10, where the plain sample series and that with one thin CFC exhibited well-distributed deformations.

The low density of fibers in the samples with one thin CFC allowed molten TPU to penetrate the fiber layer, encapsulating the fibers inside the matrix and enhancing the samples’ mechanical properties, also affecting their electrical properties. The electrical properties of the series with one thin CFC were governed by the TPU’s mechanical deformations, resulting in a nonlinear relationship with the average resistance, as shown in Figure 7a. At 10–40%, tensile deformations—the one thin CFC encapsulated inside the TPU matrix—the electrical connection between carbon fibers was maintained, and this was improved by the fibers’ length (25.4 mm). At 50% tensile deformation, the fibers partially detached from each other, increasing the resistance range values, as shown in Figure 7a, and causing scattering, as shown in Figure 8a. Furthermore, the great increase in resistance could be caused by the random orientation of the fibers and the local thickness variations of the thin CFC, which can make parts of CFC more electrically sensitive to mechanical deformations.

#### 4.2.2. Sample Series with Two Thin CFCs

In the sample series with two thin CFCs, the combination of the TPU matrix and CFCs was mechanically balanced, which can also be seen in the series’ good electrical properties. Compared with the samples with one thin CFC, the stiffness of the samples with two thin CFCs doubled (Figure 4a), and the samples showed a small force fluctuation at the start of the plastic deformation phase (Figure 4b), indicating minor reorientation or tearing of the CFC layer. The series’ closely balanced mechanical properties are also shown in Figure 10a, where the CFC layer was stiff enough to form 45° failure bands in the sample, which, however, were distributed along the samples’ length, and their deformation level was only 20% higher than in the undeformed areas. The stretchability of TPU with the long length of the carbon fibers meant that the 20% deformation level of the failure bands did not negatively affect the samples’ electromechanical properties.

The electrical properties of the sample series were stable. In Figure 7b, the resistance range stayed at the same level with 30–40% tensile deformations and with 50% tensile deformations of 500–1000 Ω. This stable behavior can be explained by the local failure bands, where tensile deformations were “absorbed”. Furthermore, the two laminated CFC plies and the interface between them could form an impermeable layer; the molten TPU did not completely bind with the lower CFC ply. In that case, the lower CFC ply can move and be further subjected to higher tensile deformations. However, only a specific degree of movement can improve the electrical properties by preserving the conductive fiber network under the movement of the TPU matrix. In contrast, too great a movement can detach and reconnect the fibers and lead to unstable electrical properties, which likely occurred in the series with three thin CFCs. With these features, the series with two thin CFCs showed the most balanced and stable electrical properties, which can be considered for the development of stretchable interconnects or sensors for wearable applications.

#### 4.2.3. Sample Series with Three Thin CFCs and with One Thick CFC

In the samples with three thin CFCs and those with one thick CFC, the CFC layer was rigid, surpassing the stiffness of the TPU matrix governing the deformations in the samples. The tensile deformations oriented and tore the CFC layer, leading to uneven mechanical results and further varying the samples’ electrical properties at high levels of tensile deformation.

As shown in Figure 4, the failure of the sample series with three thin CFCs and that of the samples with one thick CFC resembled each other, although the samples consisting of three thin CFCs had about 10 N lower maximum tensile strength than the samples with one thick CFC. Moreover, as shown in Figure 10, the samples with three thin CFCs showed a 70% difference between the least and most elongated areas, whereas the samples with one thick CFC showed a deformation difference of 40%. These results indicate that the two interfaces of the thin CFCs negatively affected the matrix’s mechanical durability and restricted the transmission of forces in the CFC layer.

During 3D printing, molten TPU likely cannot penetrate deeply into the CFC layer, although decreasing resistance due to the compression effect of TPU can be observed. The CFC and the infill area were encapsulated inside the sample, but CFC adhered only on one side, affecting the samples’ mechanical and electrical properties. This partial adherence promoted the effect of the interface areas in the CFC layers, allowing them to act as the weaker points for failures and resulting in the lower tensile properties of the samples with three thin CFCs compared to those with one thick CFC, as shown in Figure 4.

Among the entire sample series, the electrical properties of the series with three thin CFCs were the lowest in regard to resistance. The samples showed low and predictable resistance values, as shown in Figure 3, with the two interface areas between the three thin CFCs making the CFC layer conductivity very even. Still, this sample series could only sustain 10–20% tensile deformations before random failures occurred, as shown in Figure 7c and Figure 8c. Depending on the application, however, this can still be sufficient, since each sample must be subjected to an external load of approximately 100 N before reaching higher deformation levels, enabling one to use structures with three thin CFCs in low-level stretchable electronics for wearable sensor pads.

The electrical behavior of the sample series with one thick CFC and that with one thin CFC showed similar features. The difference was that the thick CFC was thicker and stiffer, making it more conductive at lower tensile deformations. However, after the breaking point, the samples exhibited higher resistance ranges because of the 45° failure bands. In fact, distributed local elongation points can orient the fibers in the tensile direction, even after thousands of cycles—whenever a failure band is formed—which can cause the resistance range to decrease, as in Figure 8d.

### 4.3. Sensor Properties of the Samples

Figure 9 shows the use of the already cyclically tested sample as a sensor. Despite the fact it had been elongated up to 50% 10,000 times, the sample exhibited a repeatable resistance curve at 10% elongation. The practical dynamic range was at least 10%, and the sensitivity was good, at approximately 10 Ω per 1% change of the full scale (~200 Ω/mm). Furthermore, the low amount of noise, drift, and the small nonlinearity error enables the application of the matrix in sensors. However, its high hysteresis decreases its accuracy and could limit its use.

In the sensor testing, the properties of the TPU matrix and sample dimensions also likely affected the resistance values. At the start of the testing process, the sample exhibited similar drift values to those of the cyclic test samples (Figure 5), which stabilized when the number of cycles increased. Fast mechanical deformations can cause dynamic errors; therefore, the 3D-printed matrix with the current dimensions exhibits the best performance when measuring slow changes.

## 5. Conclusions

Adding CFCs into the 3D printing process of a deformable TPU matrix represents a new way to make electrically conductive, stretchable, and wearable 3D-printed electronic devices. This method differs from current methods due to the possibility of creating structures with stretchability, excellent conductivity, printing accuracy, and isotropic layers. After studying the sample series during their fabrication phase, with single pull-up tests, electromechanical cyclic tests, and finally by analyzing them with DIC, the benefits of the addition of CFCs into the 3D-printed deformable matrix are obvious.

The results showed that the TPU matrix with just one nominally 53-µm-thin CFC layer provided 1500 S/m of conductivity—a result which has not been achieved with other carbon additives in the FFF process. When the thickness and number of interface areas were increased, the resistance of the CFC layers decreased to 5 Ω/10 mm, which has been conventionally achieved in the stretchable electronics field only with the use of metal-based materials.

The electromechanical properties of the CFC samples varied according to the thickness and structure of the CFC pieces, enabling versatile applications. The use of a thin and permeable CFC piece could support more stable and durable mechanical features, enabling the structure to deform based on the properties of the 3D-printed TPU matrix. The thick and multilayered CFC piece exhibited better electrical properties with low resistance and good mechanical stability, and the CFC’s stiffness governed the structure’s deformation. Between these extremes, the sample series with two thin CFCs and one interface area between the CFC plies demonstrated more balanced electromechanical properties. Small 45° failure bands with long carbon fibers absorbed tensile deformations while maintaining stable electrical properties.

These results show the potential of this novel production method to create 3D-printed stretchable electronics that can withstand high levels of deformation in a single pulling experiment and in numerous cycles. The use of one thin CFC increased the stiffness of the elastic phase but did not affect the plastic phase of 3D printed samples. The use of a high amount of CFCs increased the stiffness considerably and resulted in an irregular plastic phase, in which a 20% force decline was possible. In the cyclic testing, the samples with one thin CFC or two thin CFCs layers could reach even higher elongation than that measured at 50% for 10,000 cycles. This fabrication approach is easy to incorporate into current 3D printing practices, enabling the preparation of 3D-printed stretchable and wearable electronics with commercially available materials and methods. Varying the thickness and number of CFCs added into the 3D-printed matrix allows one to optimize the structures’ properties for different purposes. Additive-manufactured deformable CFC electronics can be used, for example, in sensors, interconnects, and circuit boards.

The use of CFCs inside 3D-printed structures has advantages, for example, using one thin CFC in several layers in FFF so that every printing layer adheres to only one layer of a thin CFC can be implemented to make highly reinforced structures that elongate steadily and which have intermediate electrical conductivity. Moreover, the CFCs on multiple layers can be shaped differently, enabling simple 3D PCBs that are stretchable and wearable. However, the manufacturing parameters of 3D-printed TPU matrices and CFCs of differing thicknesses require further optimization for sensor applications. Furthermore, the placement method of CFCs needs to be automated, for instance, with the use of a modified pick-and-place machine.

## Figures and Tables

**Table 1:** Studied CFC compositions based on the manufacturer’s data.

Series	Thickness (µm)	Weight (g/m^2^)	N of Layers	Grade
1	-	-	0	-
2	53	6.8	1	800015i
3	106	13.6	2	800015i
4	159	20.3	3	800015i
5	153	17	1	800020i

**Table 2:** Studied CFC compositions based on the manufacturer’s data, and the target and measured average dimensions of the sample series.

Series	Average Width (mm)	Average Thickness (mm)	Average Area (mm^2^)
Target dimensions	10	1	10
Without CFC	9.97	1.02	10.15
1 thin CFC (53 µm)	10.09	1.03	10.41
2 thin CFCs (106 µm)	10.12	1.04	10.56
3 thin CFCs (159 µm)	10.21	1.06	10.83
1 thick CFC (153 µm)	10.11	1.04	10.49

## Data Availability

Not applicable.

## References

[1] Singh S., Singh G., Prakash C., Ramakrishna S. (2020). Current status and future directions of fused filament fabrication. J. Manuf. Process..

[2] Hohimer C.J., Petrossian G., Ameli A., Mo C., Pötschke P. (2020). 3D printed conductive thermoplastic polyurethane/carbon nanotube composites for capacitive and piezoresistive sensing in soft pneumatic actuators. Addit. Manuf..

[3] Kim K., Park J., Suh J., Kim M., Jeong Y., Park I. (2017). 3D printing of multiaxial force sensors using carbon nanotube (CNT)/thermoplastic polyurethane (TPU) filaments. Sens. Actuators A Phys..

[4] Reyes C., Somogyi R., Niu S., Cruz M.A., Yang F., Catenacci M.J., Rhodes C.P., Wiley B.J. (2018). Three-Dimensional Printing of a Complete Lithium Ion Battery with Fused Filament Fabrication. ACS Appl. Energy Mater..

[5] Ahrendt D., Romero Karam A. (2020). Development of a computer-aided engineering–supported process for the manufacturing of customized orthopaedic devices by three-dimensional printing onto textile surfaces. J. Eng. Fibers Fabr..

[6] Uysal R., Stubbs J.B. (2019). A New Method of Printing Multi-Material Textiles by Fused Deposition Modelling (FDM). TEKSTILEC.

[7] Salo T., Halme A., Lahtinen J., Vanhala J. (2020). Enhanced stretchable electronics made by fused-filament fabrication. Flex. Print. Electron..

[8] Stuart T., Kasper K.A., Iwerunmor I.C., McGuire D.T., Peralta R., Hanna J., Johnson M., Farley M., LaMantia T., Udorvich P. (2021). Biosymbiotic, personalized, and digitally manufactured wireless devices for indefinite collection of high-fidelity biosignals. Sci. Adv..

[9] Ly S.T., Kim J.Y. (2017). 4D printing–fused deposition modeling printing with thermal-responsive shape memory polymers. Int. J. Precis. Eng. Manuf. Technol..

[10] Tekinalp H.L., Kunc V., Velez-Garcia G.M., Duty C.E., Love L.J., Naskar A.K., Blue C.A., Ozcan S. (2014). Highly oriented carbon fiber-polymer composites via additive manufacturing. Compos. Sci. Technol..

[11] Zhang J., Yang B., Fu F., You F., Dong X., Dai M. (2017). Resistivity and Its Anisotropy Characterization of 3D-Printed Acrylonitrile Butadiene Styrene Copolymer (ABS)/Carbon Black (CB) Composites. Appl. Sci..

[12] Wei X., Li D., Jiang W., Gu Z., Wang X., Zhang Z., Sun Z. (2015). 3D Printable Graphene Composite. Sci. Rep..

[13] Jahangir M.N., Billah K.M.M., Lin Y., Roberson D.A., Wicker R.B., Espalin D. (2019). Reinforcement of material extrusion 3D printed polycarbonate using continuous carbon fiber. Addit. Manuf..

[14] Al-Saleh M.H., Sundararaj U. (2011). Review of the mechanical properties of carbon nanofiber/polymer composites. Compos. Part A Appl. Sci. Manuf..

[15] Forintos N., Czigany T. (2019). Multifunctional application of carbon fiber reinforced polymer composites: Electrical properties of the reinforcing carbon fibers—A short review. Compos. Part B Eng..

[16] Li Y., Huang X., Zeng L., Li R., Tian H., Fu X., Wang Y., Zhong W.H. (2019). A review of the electrical and mechanical properties of carbon nanofiller-reinforced polymer composites. J. Mater. Sci..

[17] Tzounis L., Petousis M., Grammatikos S., Vidakis N. (2020). 3D printed thermoelectric polyurethane/multiwalled carbon nanotube nanocomposites: A novel approach towards the fabrication of flexible and stretchable organic thermoelectrics. Materials.

[18] Spoerk M., Savandaiah C., Arbeiter F., Traxler G., Cardon L., Holzer C., Sapkota J. (2018). Anisotropic properties of oriented short carbon fibre filled polypropylene parts fabricated by extrusion-based additive manufacturing. Compos. Part A Appl. Sci. Manuf..

[19] Yin L., Tian X., Shang Z., Wang X., Hou Z. (2019). Characterizations of continuous carbon fiber-reinforced composites for electromagnetic interference shielding fabricated by 3D printing. Appl. Phys. A..

[20] Wang Z., Luan C., Liao G., Yao X., Fu J. (2019). Mechanical and self-monitoring behaviors of 3D printing smart continuous carbon fiber-thermoplastic lattice truss sandwich structure. Compos. Part B Eng..

[21] Hu Q., Duan Y., Zhang H., Liu D., Yan B., Peng F. (2018). Manufacturing and 3D printing of continuous carbon fiber prepreg filament. J. Mater. Sci..

[22] Yu T., Zhang Z., Song S., Bai Y., Wu D. (2019). Tensile and flexural behaviors of additively manufactured continuous carbon fiber-reinforced polymer composites. Compos. Struct..

[23] Türk D.-A., Ebnöther A., Zogg M., Meboldt M. (2018). Additive Manufacturing of Structural Cores and Washout Tooling for Autoclave Curing of Hybrid Composite Structures. J. Manuf. Sci. Eng..

[24] Yao X., Luan C., Zhang D., Lan L., Fu J. (2017). Evaluation of carbon fiber-embedded 3D printed structures for strengthening and structural-health monitoring. Mater. Des..

[25] Falco A., Petrelli M., Bezzeccheri E., Abdelhalim A., Lugli P. (2016). Towards 3D-printed organic electronics: Planarization and spray-deposition of functional layers onto 3D-printed objects. Org. Electron..

[26] Abdelhalim A., Abdellah A., Scarpa G., Lugli P. (2013). Fabrication of carbon nanotube thin films on flexible substrates by spray deposition and transfer printing. Carbon N. Y..

[27] Fieber L., Evans J.D., Huang C., Grant P.S. (2019). Single-operation, multi-phase additive manufacture of electro-chemical double layer capacitor devices. Addit. Manuf..

[28] ACP Composites (2014). Carbon Fiber Tissue. Product Data Sheet. https://store.acpcomposites.com/carbon-fiber-veil?quantity=1&weight=1.

[29] Hagberg J., Leijonmarck S., Lindbergh G. (2016). High Precision Coulometry of Commercial PAN-Based Carbon Fibers as Electrodes in Structural Batteries. J. Electrochem. Soc..

[30] Persons A.K., Ball J.E., Freeman C., Macias D.M., Simpson C.L., Smith B.K., Burch V.R.F. (2021). Fatigue testing of wearable sensing technologies: Issues and opportunities. Materials.

[31] Guadagno L., Vertuccio L., Naddeo C., Raimondo M., Barra G., De Nicola F., Volponi R., Lamberti P., Spinelli G., Tucci V. (2019). Electrical current map and bulk conductivity of carbon fiber-reinforced nanocomposites. Polymers.

